# Label-Free Biosensor Using a Silver Specific RNA-Cleaving DNAzyme Functionalized Single-Walled Carbon Nanotube for Silver Ion Determination

**DOI:** 10.3390/nano8040258

**Published:** 2018-04-20

**Authors:** Hui Wang, Yang Liu, Gang Liu

**Affiliations:** 1 Key Laboratory of Modern Precision Agriculture System Integration Research, Ministry of Education and Key Laboratory of Agricultural Information Acquisition Technology, Ministry of Agriculture China Agricultural University, Beijing 100083, China; wanghui_lunwen@163.com; 2 School of Electronic and Information Engineering, Xi’an Jiaotong University, Xi’an 710049, China; yliu@sei.xjtu.edu.cn

**Keywords:** field effect transistor, single-walled carbon nanotubes, silver ion, biosensor, DNAzyme

## Abstract

Silver, a very common heavy metal, has been employed in electronics, medicine, jewelry, and catalysis due to its excellent chemical and physical characteristics. Silver-containing wastes can cause environmental pollution, so it is vital to monitor the Ag(I) concentration. Here, a label-free biosensor was developed for the Ag(I) detection, which used single-walled carbon nanotubes/field effect transistor (SWNTs/FET) to functionalize with a specific DNAzyme, containing an Agzyme and a complementary strand DNA (CS-DNA) embedded an RNA-base. The CS-DNA was covalently immobilized on the SWNTs’ surface through peptide bonds, and then combined with the Agzyme. When Ag(I) was bound with the Agzyme, the CS-DNA can be cleaved at the RNA site efficiently. The cleaved DNAzyme induced a remarkable change in the electrical conductivity of SWNTs. The performances of DNAzyme/SWNTs/FET were investigated using different spectroscopy and electrochemical methods. Under the optimized parameters, DNAzyme/SWNTs/FET presented a high sensitivity and selectivity towards Ag(I), in which the linear response range is 10 pM to 10^6^ pM and the limit of detection is 5 pM(S/N = 3). Additionally, the prepared biosensor was applied to measure the Ag(I) concentration in the water sample with good results.

## 1. Introduction

Heavy metal pollution [1,2] has attracted attention in many developing countries as heavy metals have acute toxicity that threatens human health [3,4]. Silver [5], a common heavy metal, was increasingly used in ornaments [6], food packaging [7], medical instruments [8], plastics [9], refrigerators [10], electronic equipment [11], and dental fillings [12], which was mainly ascribed to its thermal, electrical, optical, catalytic, and unique antibacterial properties [13,14]. With unreasonable mining and waste management, untreated pollutants containing silver and its compounds were discharged into the surrounding. According to statistics, silver emissions from industrial wastes were approximately 2,500 tons every year, of which 80 tons entered surface waters and 150 tons were released into the sludge of wastewater treatment plants [14,15]. That led the level of silver to exceed the background levels in soil and water [16]. However, silver ions (Ag(I)) were one of the most toxic forms [17], secondary to only mercury. Ag(I) had high affinity with biological molecules because it can easily bind with the sulfur atoms of the methionine (-SCH) [18] and sulfhydryl groups (-SH) of cysteine [19] to form a stable complex, which will affect the activity of proteins and enzymes. If humans ingested the polluted water and food for a long period, Ag(I) would be accumulated in human body. It might probably cause serious diseases with various symptoms, including stomach pains, breathing problems, lung and throat irritation, a blue-gray discoloration of the skin, and mild allergic reactions [20,21]. To guarantee human health, the United States Environmental Protection Agency (USEPA) required the Ag(I) concentration in drinking water to not exceed 0.10 ppb [22].

Therefore, an approach to quickly identify and measure the Ag(I) concentration was crucial to evaluate the Ag(I) pollution in the water environment. Many regular methods were used for the Ag(I) determination, such as atomic absorption/emission spectroscopy (AAS/AES) [23,24], cold vapor atomic fluorescence spectrometry (CVAFS) [25], ultraviolet-visible (UV–VIS) spectroscopy [26], inductively-coupled plasma mass spectrometry (ICP-MS) [27], and fluorescence spectroscopy [28]. These methods were highly selective and sensitive. Nonetheless, the devices were too expensive and bulky, and also need skilled workers to perform complicated pretreatments and operate the sophisticated instrument. Additionally, these instruments were difficult to use on-site/in-site. The reasons mentioned above limited their popularization and application in daily life. Electrochemical methods were economic and convenient technologies, which only needed a small device to record the electronic signals, overcoming the use of expensive signal-transforming instruments. For recent decades, electrochemical sensors and biosensors regained attention in many fields due to the development of chemical and biological materials. These new materials provided an innovative mechanism to improve the performance of sensors or biosensors.

Over the past years, many biosensors were developed and applied to Ag(I) detection. Some of them mainly used a specific deoxyribonucleic acid (DNA) because Ag(I) can specially bind with two cytosines (C) to form a structure “C-Ag-C” [29,30]. Liujiao Bian and co-workers [31] designed a single-labeled fluorescent oligonucleotide probe using C-enriched DNA in which the structural switch of the probe DNA can cause fluorescence quenching to detect Ag(I). The C-based biosensors have high sensitivity and selectivity, but the response time was too long. Zhou’s group [32] proposed an ultrasensitive assay using peptide-AuNPs for Ag(I) detection in rivers and lakes. Until now, many DNAzymes had been designed to determine different metal ions, including Na(I), Ag(I), Pb(II), Cr(II), Cu(II), Hg(II), Zn(II), and UO_2_(II). In 2016, Liu et al. [33] reported an Ag(I)-specific RNA-cleaving DNAzyme named Ag10c. This DNAzyme had high selectivity for Ag(I) over other metal ions with the catalytic rate 0.41 min^−1^ at pH 7.5. His group further investigated the biophysical insights of DNAzyme using 2AP fluorescence as a probe in the following years. They also established some fluorescent biosensors for Ag(I) sensing, which presented high sensitivity and a short response time compared with C-based biosensors. In the following years, many fluorescence biosensors using the DNAzyme had been designed for Ag(I) detection. However, all of the fluorescence biosensors had background fluorescence that made it difficult for these biosensors to identify a very small signal change, especially at low Ag(I) concentration. Additionally, the detection accuracy can be affected by impurity sample.

**Figure 1:**
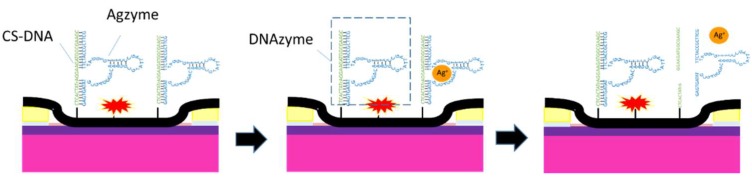
Schematic illustration of the detecting mechanism.

To improve the sensitivity and accuracy, we developed a field-effect transistor (FET) biosensor using single-walled carbon nanotubes (SWNTs) and the Ag(I) specific RNA-cleaving DNAzyme. As shown in Figure 1, the mechanism of this biosensor was that one Agzyme initially bound with one Ag(I) in a short time, and then this combination can cleave the complementary strand DNA embedded in an RNA-base. Due to the excellent semiconductor properties of SWNTs, the structural change of DNAzyme led to an increase in the carrier concentration in SWNTs, which will further affect the conductivity of the biosensor. Based on the electrical conductivity change, the relationship was established between the Ag(I) concentrations and the relative resistances (“mol/L” was abbreviated as “M” in this article.).

## 2. Materials and Methods

### 2.1. Chemicals and Materials

Metal salts (KNO_3_, NaNO_3_, Pb(NO_3_)_2_, Cd(NO_3_)_2_, NaNO_3_, Mg(NO_3_)_2_, Fe(NO_3_)_3_, Zn(NO_3_)_2_, Cr(NO_3_)_3_), 6-mercapto-1-hexanol (MCH, 97%), dimethyl formamide (DMF), and ethanolamine were purchased from Sigma Aldrich (Hampton, NH, USA). 1-Pyrenebutanoic acid succinimidyl ester (PBASE) was bought from Invitrogen Inc. (Carlsbad, CA, USA). 3-Aminopropyltriethoxysilane (APTES, 99%) and ethanolamine (EA, 99%) were purchased from Acros Organics (Hampton, NH, USA). A homogeneous dispersion of single-walled carbon nanotubes (0.01 mg/mL, 95% semiconducting) was provided by Nano-Integris Inc. (Gaomi, Shandong, China). Tween 20 was offered by Bio-Rad. The rest of chemical reagents not mentioned here were of analytical reagent grade and were used without further purification. A stock solution of Ag(I) was prepared by dissolving AgNO_3_ with 0.1% HNO_3_. 2-(*N*-morpholino)ethane sulfonic acid (MES) and 3-(*N*-morpholino) propane sulfonic acid (MOPS) were from Fisher Scientific (Hampton, NH, USA). Milli-Q water (resistance > 18.2 MΩ) without any further purification was used directly throughout the experiments.

Oligonucleotides were synthesized by the standard solid phase technique using a fully-automated DNA synthesizer and HPLC purified by Shanghai Sangon Biotechnology Co., Ltd. (Shanghai, China). The concentrations of DNA were determined using the 260 nm UV absorbance. The sequences were:

CS DNA: 5′-(NH_2_)-CTCACTATrAGGAAGATGGCGAAGC-3′

CP DNA: 5′-(NH_2_)-CTCACTATAGGAAGATGGCGAAGC-3′

Agzyme: 5′-GCTTCGCCATCTTTAGGTGATTTCCACGATTATGCGGAAACAGGGCAGCGTATAGTGAG-3′

### 2.2. Apparatus

The characteristics of the device were investigated by scanning electron microscope (SEM), Raman, UV–VIS spectrometer and current-voltage (I–V) measurement. The images of the scanning electron microscope were collected using a Zeiss Leo SUPRA 55 (Zeiss, Braunschweig, Germany). Raman spectrometer with imaging microscope was Dilor XY Laser Raman (514 nm diode and Ar ion lasers) to record the Raman spectra (HORIBA Ltd., Paris, France). All of the I–V measurements were obtained using a Keithley 2636 (Mansfield, TX, USA) semiconductor parameter analyzer. The UV spectrum was collected with a Beckman DU800 UV–VIS spectrophotometer (Beckman Coulter, Inc. Pasadena, CA, USA).

### 2.3. Fabrication of SWNTs-FET

A highly-doped p-type silicon wafer with 100 nm thick thermal oxide (SiO_2_) was selected as the substrate. The substrate was cleaned using acetone and isopropanol, the photoresist was spun on its surface, and the single gap structure was written through standard lithographic patterning. After baking, a 20 nm-thick Cr layer and a 180 nm-thick Au film were deposited on the surface, respectively. The structure was shown in Figure S1A. The length and width of the gap between the drain and source in Figure S1B were 10 μm and 10 μm, respectively. The deposited wafer was immersed in acetone overnight to remove the residual of Cr and Au.

SWNTs were functionalized on FET through the following steps. First, the chip was cleaned successively with acetone, isopropanol, and ammonium hydroxide. Second, the cleaned chip was incubated in APTES for 60 min and rinsed with sufficient Milli-Q water. Third, SWNTs solution was used to cover the area between the source and the drain under the high humidity condition for 60 min, followed by annealing in air at 250 °C. The morphology of SWNTs-FET is shown in Figure S1C.

### 2.4. Immobilization

**Figure 2:**
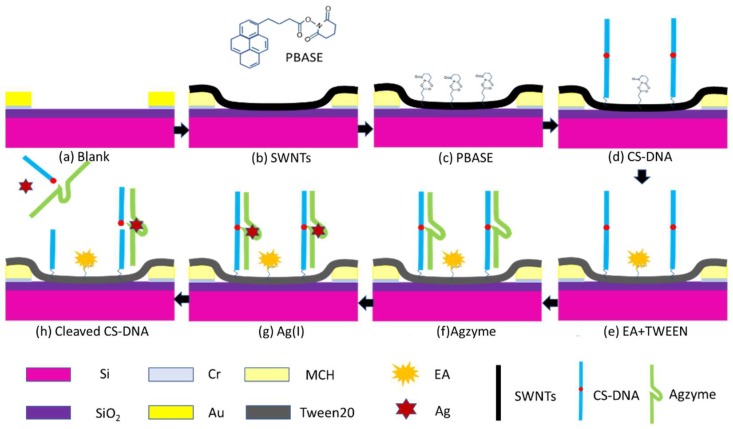
Schematic illustration of SWNT-based biosensor surface functionalization with different materials (SWNTs, MCH, PBASE, CS-DNA, EA, TWEEN20, and Agzyme) and Ag(I) is used to induce cleavage of the DNAzyme.

In order to immobilize DNAzyme, SWNTs/FET was treated as the following steps in Figure 2. SWNTs/FET was immersed in 10 mM MCH solution for 30 min to block the gold surface, and then treated with 6 mM PBASE in dimethylformamide for 60 min at room temperature, which was rinsed with Milli-Q water to remove the unreacted probe after each step. Next, the chip was incubated with 100 μM CS-DNA overnight at 4 °C, which immobilized with SWNTs through the peptide bond between the amine at the 5′ end and the ester groups of PBASE. The excess ester groups of PBASE were blocked using 0.1 mM EA solution, and the bare SWNTs were blocked using 0.1% Tween 20 to prevent non-specific binding to SWNTs. Finally, the CS-DNA on the SWNTs’ surface was hybridized with 100 μM Agzyme for 2 h to form the DNAzyme.

### 2.5. Sensing Protocol

For I–V measurements, the voltage between the drain and the source (V_DS_) was ramped from −0.2 V to +0.2 V with the gate bias set to 0 V (V_G_ = 0 V), and the current was recorded at the same time. The resistance value (R) was calculated through Ohm’s law (R = U/I).

To determinate the Ag(I) concentration, DNAzyme/SWNTs/FET was covered with MOPS solution and the I–V values were recorded. After that, the biosensor was immersed in the test solution for 3 min, and then rinsed with sufficient MOPS buffer solution to remove the Ag(I) residues. Moreover, the biosensor was covered with MOPS solution again and measured the I–V. The relative change of resistance is defined as follow:

∆R/R = (R_0_ − R)/R_0_ × 100%

where R_0_ was the initial resistance of DNAzyme/SWNTs/FET before exposure to the Ag(I) solution, and R was the resistance of DNAzyme/SWNTs/FET after exposure to the Ag(I) solution.

## 3. Results

### 3.1. Characterization

**Figure 3:**
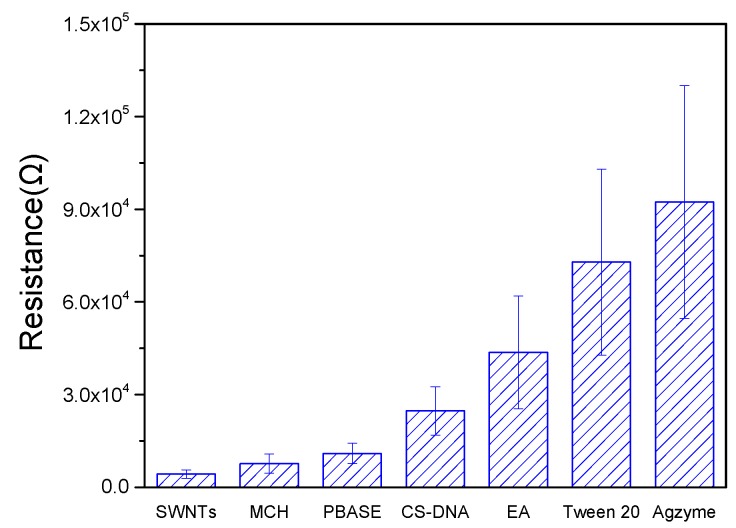
Changes in resistance after FET functionalized with different materials (SWNTs, MCH, PBASE, CS-DNA, EA, TWEEN20, and Agzyme) at V_DS_ = −0.02 V.

The I–V measurements in Figure S1 were collected after the SWNTs/FET was modified by MCH, PBASE, CS-DNA, EA, Tween 20, and Agzyme. Comparing the I–V curve of SWNTs/FET, the slopes of the other curves continued to decrease after each step, indicating that the electrical conductivity of SWNTs-FET decreased. As shown in Figure 3, the resistance of SWNTs-FET was about 4.2 KΩ. When SWNTs/FET was incubated in MCH, the resistance change was very small because MCH only bound with gold through Au-S bonds to form a nonconductive membrane. The resistances increased dramatically after SWNTs functionalized with PBASE, CS-DNA, EA, Tween 20, and Agzyme, which were mainly attributed to π–π interaction between SWNTs and pyrene moieties in PBASE, electron donation from amine labeled CS-DNA, EA, Tween 20, and Agzyme resulting in a decrease of the charge carrier concentration of the p-type SWNTs, and/or electron scattering from these molecules.

**Figure 4:**
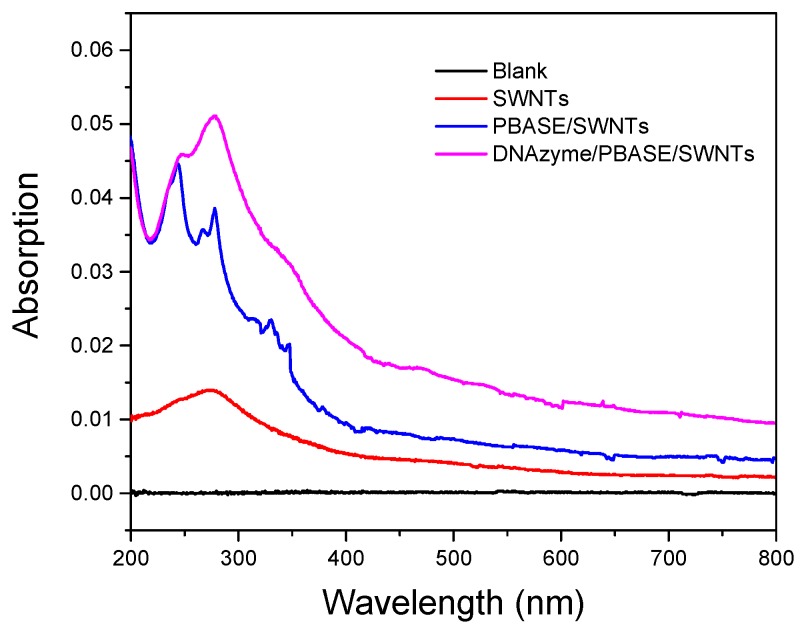
UV–VIS absorption spectra of quartz substrate functionalized with SWNTs, PBASE, and DNAzyme.

To illustrate the UV–VIS spectra of SWNTs before and after being functionalized with PBASE and DNAzyme, we chose a quartz plate to replace the silicon substrate due to the optical transparency. As shown in Figure 4, the spectrum of blank quartz was selected as the background, so the absorbance was zero. When the quartz was modified by SWNTs, the absorbance intensity of the whole spectra range enhanced, and an absorption band was found at 275 nm, illustrating SWNTs was immobilized on the quartz’s surface [34,35]. There were three absorbance bands revealed on the curve of SWNTs/PBASE, which was consistent with the previous papers [36]. This was because the pyrene ring was attached to the SWNTs’ surface through the π–π interaction. After SWNTs/PBASE was functionalized with CS-DNA and Agzyme, the absorption band was enhanced, which might be the superposition of all the absorption bands.

**Figure 5:**
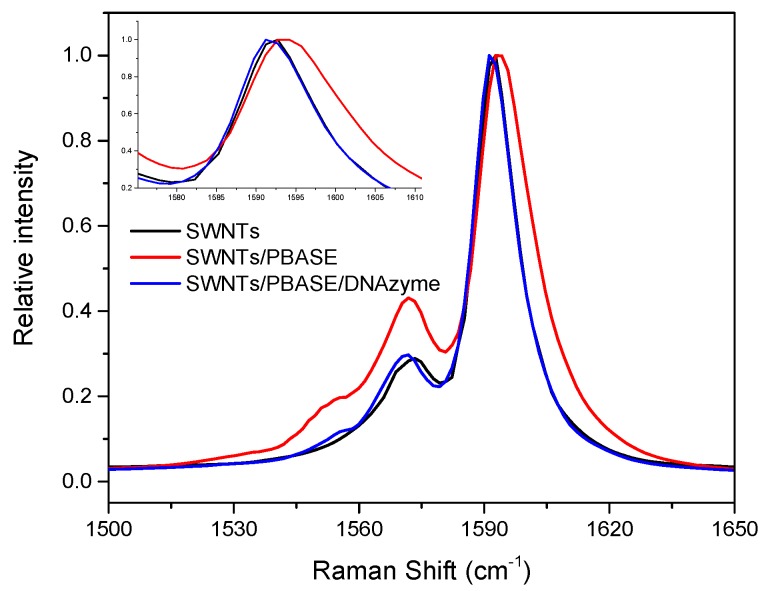
Raman spectra of SWNTs functionalized with PBASE and DNAzyme.

Figure 5 shows the Raman spectrum of SWNTs functionalized with PBASE and DNA using 514 nm laser excitation. The G peak of SWNTs was located at 1592 cm^−1^. After PBASE was functionalized on the SWNTs’ surface, the G band shifted to the right at 1594 cm^−1^. A possible reason for the G band shift about 2 cm^−1^ was that PBASE bound with SWNTs that changed the charge transfer of SWNTs. The G band of DNAzyme/PBASE/SWNTs shifted to the opposite direction about 3 cm^−1^, indicating that DNAzyme had attached to the SWNTs’ surface.

### 3.2. Optimization

To improve the performance, some parameters were optimized. We first studied the effect of the base type of the cleavage junction using the DNAzyme/SWNTs/FET to detect the three Ag(I) concentrations (100 pM, 100 nM, 100 μM) in Figure S2. When the ‘rA’ base was replaced by “A”, the relative resistances were no change in the presence of three different Ag(I) concentrations. Therefore, it demonstrated that the site of Ag(I)-cleaved CS-DNA occurred at the position of “rA”.

**Figure 6:**
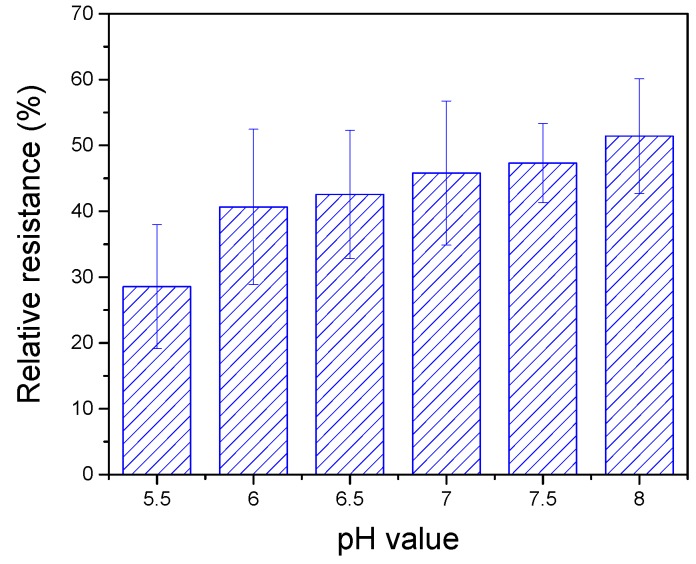
Effect of pH value on the performance of the biosensor with 10 nM Ag(I) concentration at V_G_ = 0 V.

Figure 6 shows the relative resistances of DNAzyme/SWNTs/FET affected by the pH of the EPOS solution. The relative resistance had a positive correlativity with pH values, which meant that the higher pH might improve the efficiency of Ag-cleavage yields. The reason was the deprotonation of the 2′-OH of the RNA base, making it a better nucleophile [33]. However, if the buffer solution was alkaline, the Ag(I) concentration decreases and precipitates. Therefore, a pH of 7.5 was used in the following experiments.

**Figure 7:**
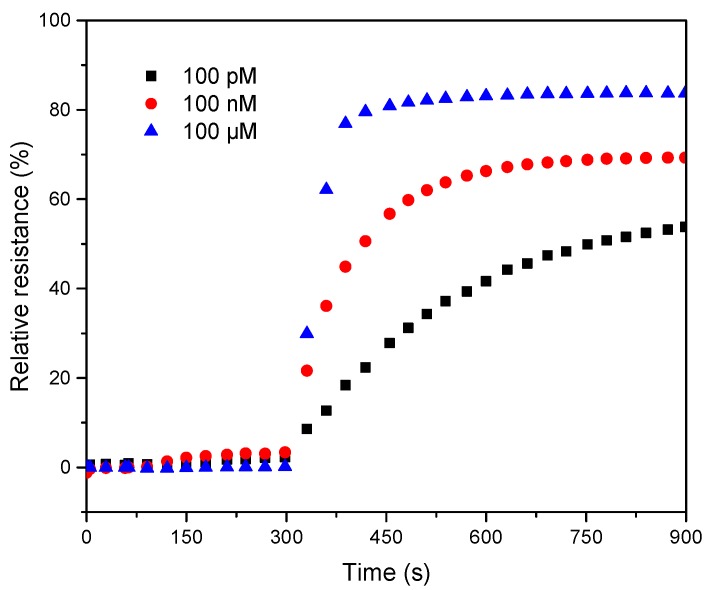
The effect of incubation time on the performance of the biosensor with the three Ag(I) concentrations (100 pM, 100 nM, 100 μM) at V_DS_ = 0.1 V and V_G_ = 0 V.

To obtain a short response time, three different concentrations (100 pM, 100 nM, 100 μM) of Ag(I) were selected. DNAzyme/SWNTs/FET was incubated in pH 7.5 MOPS without Ag(I) during the first 5 min, and then incubated in the different Ag(I) concentrations for 10 min. During the incubation, the currents were measured every 30 s. Figures S3 and S4 show the currents and resistances at different points in time. After DNAzyme/SWNTs/FET was exposed to the Ag(I) solution, the currents increased dramatically in the first several minutes and then begin to flatten. The signal intensity correlated strongly with the incubation time in the beginning. Comparison of the three curves in Figure 7, the growth rates of relative resistance for 100 nM and 100 μM Ag(I) concentration were fast in the first 150 s and leveled off after 180 s. Therefore, 3 min was chosen as the incubation time.

**Figure 8:**
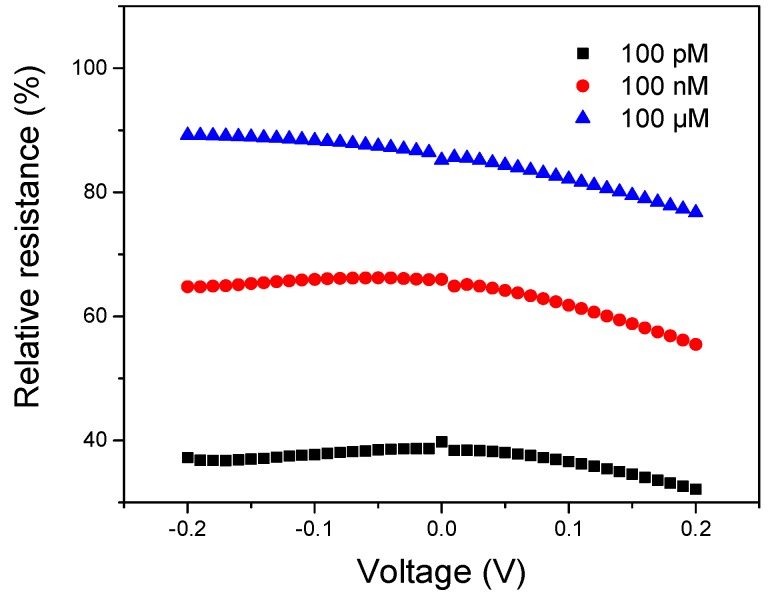
Effect of the V_DS_ ranging from −0.2 V to 0.2 V on the performance of the biosensor with the three Ag(I) concentrations (100 pM, 100 nM, 100 μM) at V_G_ = 0 V.

To demonstrate the feasibility that the relative resistance can decrease the detection signal error, DNAzyme/SWNTs/FET was measured three different concentrations (0 pM, 100 pM, 100 nM, 100 μM) of Ag(I). Figure S5 shows the I–V curves, which the voltage was ranging from −0.2 V to +0.2 V. with the increasing Ag(I) concentration, the currents become larger. However, the currents were not linear with voltages. The resistance values at different voltages were shown in Figure S6, which also exhibited nonlinearity. The reason was that alkaline buffer solution equated approximately to a negative base voltage which can cause the carrier mobility change of SWNTs. After calculating the relative resistance, the values at different voltages in Figure 8 are approximately constant, illustrating that the relative resistance can decrease the detection signal error. Based on the energy saving and stability, −0.02 V was chosen for this experiment.

### 3.3. Analytical Performance

**Figure 9:**
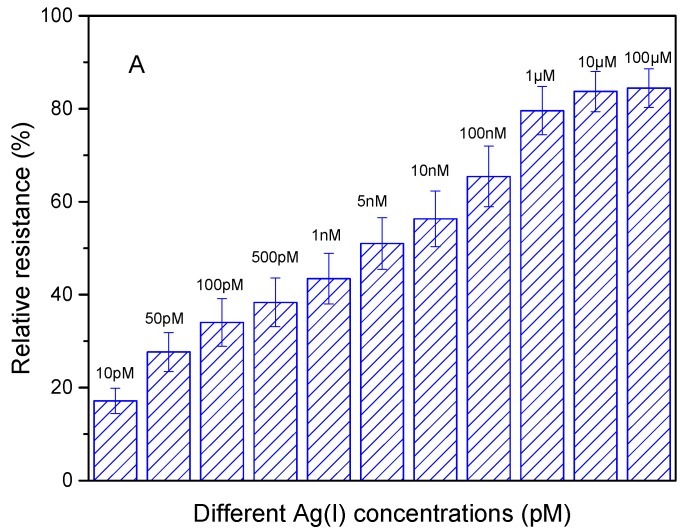

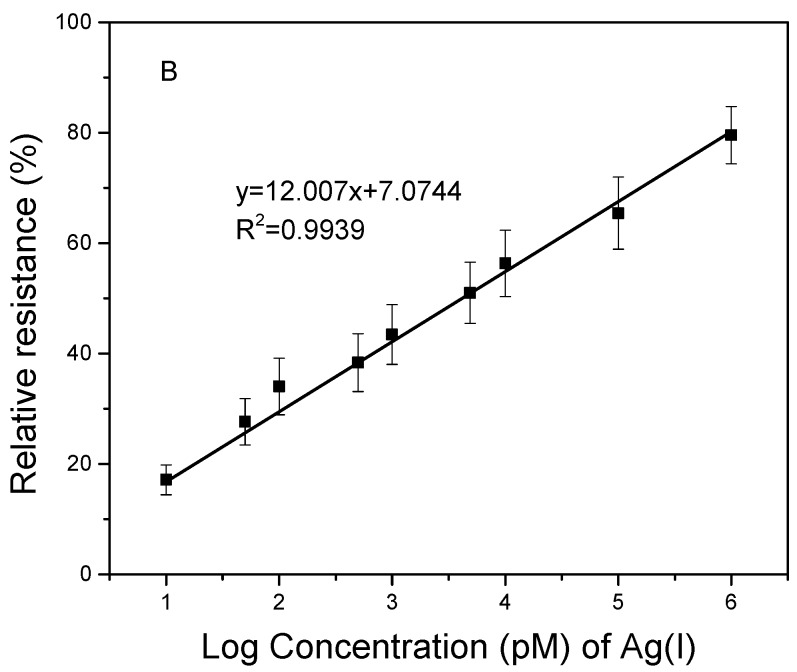
The determination of Ag(I). (**A**) Relative resistance change after exposed to different concentrations of 10, 50, 100, 500, 1000, 5000, 10,000, 100,000, 1,000,000, 10,000,000, and 100,000,000 pM. (**B**) The response linearity between the relative resistance and the logarithm of Ag(I) concentration.

To obtain the linear range and the limit of detection, the analytical performance of DNAzyme/SWNTs/FET was further studied under the optimal conditions discussed above. Briefly, the biosensor was exposed to various standard concentrations of Ag(I) solutions in 10 mM MOPS buffer at pH 7.5 for 3 min. After that, the biosensor was rinsed and covered with MOPS solution to measure the current. After calculating the relative resistance, the results were shown in Figure 9A, which were increasing with the Ag(I) concentrations ranging from 10 pM to 100 μM. However, the relative resistance presented a linear relation with the logarithm of Ag(I) concentrations during 10 pM to 1 μM. The regression equation in Figure 9B was y = 12.007 log[Ag(I)] + 7.0744 with the correlation coefficient R more than 0.99. The limit of detection was 5 pM (S/N = 3), which was substantially below the permissible Ag(I) concentration specified by the United States Environmental Protection Agency (USEPA) in drinkable water.

Table 1 shows the performance comparison of the prepared biosensor with that of previously-reported sensors. It demonstrates that the present biosensor had a broader linear range, which the detection limit was lower than other sensors. Compared to other sensors, this high-performance biosensor was much suited for the Ag(I) determination in-site.

### 3.4. Interference

**Figure 10:**
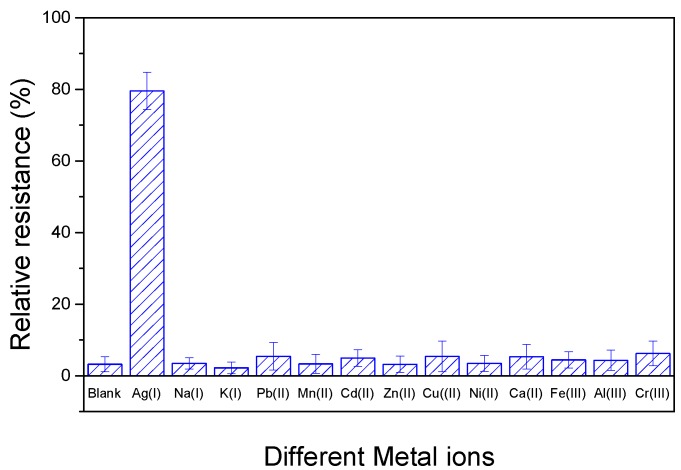
Relative resistance of the biosensor with 1 μM Ag(I) and 100 μM common metal ions. The error bars represent standard deviations based on three independent measurements.

The selectivity of DNAzyme/SWNTs/FET was evaluated by testing different monovalent, divalent, and trivalent cations that probably existed in water environment, including Na(I), K(I), Pb(II), Mn(II), Cd(II), Zn(II), Cu(II), Ni(II), Ca(II), Fe(III), Al(III), and Cr(III). Figure 10 shows the relative resistances that the biosensor exposed to 1 μM Ag(I) or 10-fold other metal ions (10 μM). We found that the relative resistances were no change after detecting most 10-fold other metal ions. However, Pb(II), Cd(II), and Cu(II) led to slight changes, but the relative errors were no more than 5%. It demonstrates that DNAzyme/SWNTs/FET had an excellent selectivity toward Ag(I).

### 3.5. Real Sample Analysis

To further evaluate the applicability of the biosensor, three water samples were collected from the river in our city. Before testing, each water sample was pretreated through the following steps. First, a water sample was filtered using a 0.22 µm membrane to remove the undissolved substance; second, the pH value of the filtered sample was adjusted to 7.5 by adding MOPS buffer solution. Subsequently, the water sample was spiked with the standard Ag(I) solution. Table 2 shows the results using the DNAzyme/SWNTs/FET and AAS. The recovery ranged from 92.45% to 105.12%, indicating that the proposed biosensor can measure the Ag(I) concentration in river water with acceptable accuracy. The reason that caused the recovery rate to be over 100% was that some coexisted metal ions or organic impurities in water samples may attach to the SWNTs’ surface. Therefore, we can confirm that the proposed biosensor had relative good applicability.

## 4. Conclusions

In summary, we developed a sensitive and selective biosensor for Ag(I) detection. This biosensor used an Ag(I)-specific DNAzyme functionalized with an SWNTs-based FET. The Ag(I) can efficiently bind with Agzyme and cleave the CS-DNA, which led to the DNAzyme’s structural switch and changed the conductivity of the SWNTs. Due to the high specificity and fast catalytic rate, the DNAzyme/SWNTs/FET showed excellent sensitivity and selectivity for Ag(I) sensing at low concentrations over other metal ions. The LOD of DNAzyme/SWNTs/FET was 5 pM, which was far below the permissible limit of silver in water.

## Figures and Tables

**Table 1:** Comparison the parameters of the prepared biosensor with other sensors for Ag(II) detection.

Sensor	Method	Liner Ranges (M)	Determination Limits (M)	Reference
OND-Ag(I)	Fluorescence	1.0 × 10^−9^~1.0 × 10^−7^	5.0 × 10^−11^	[31]
G-Quadruplex-Hemin DNAzymes	UV-vis	5.0 × 10^−8^~3.0 × 10^−6^	6.4 × 10^−8^	[37]
RBITC-AuNP	Fluorescence	1.0 × 10^−8^~7.0 × 10^−7^	4.8 × 10^−10^	[38]
SGI/C-DNA	Fluorescence	1.0 × 10^−8^~6.0 × 10^−7^	4.3 × 10^−9^	[39]
CNP-OND	Fluorescence	5.0 × 10^−10^~4.0 × 10^−7^	5.0 × 10^−10^	[40]
QF-TAMRA	Fluorescence	1.6 × 10^−11^~2.0 × 10^−8^	2.4 × 10^−12^	[41]
DNA-MSS@AuNP	SERS	1.0 × 10^−8^~1.0 × 10^−6^	1.0 × 10^−9^	[42]
DNAyme/SWNTs/FET	IV	1.0 × 10^−11^~1.0 × 10^−6^	5 × 10^−12^	This work

**Table 2:** Results of the Ag(I) recovery experiments in river water using the proposed biosensor.

Sample	Adding Ag(I)	DNAyme/SWNTs/FET	AAS	Recovery
	nM	nM	nM	%
1	-	1.23	1.17	105.12
	5	6.31		102.27
2	-	1.46	1.42	102.82
	5	6.35		98.91
3	-	0.98	1.06	92.45
	5	6.14		101.32

## Supplementary Materials

The following are available online at http://www.mdpi.com/2079-4991/8/4/258/s1, Figure S1: SEM image of SWNT networks produced by APTES-assisted assembly technique, Figure S2: Effect of the “A” base type of the Ag cleavage junction on the performance of the biosensor with the three Ag(I) concentrations (100 pM, 100 nM, 100 μM), Figure S3: The relationship between current and incubation time on the performance of the biosensor with the three Ag(I) concentrations (100 pM, 100 nM, 100 μM) at VG = 0 V, Figure S4: The relationship between resistance and incubation time on the performance of the biosensor with the three Ag(I) concentrations (100 pM, 100 nM, 100 μM) at VG = 0 V, Figure S5: The effect of the voltage ranging from −0.2 V to 0.2 V on the performance of the biosensor with the three Ag(I) concentrations (0 pM, 100 pM, 100 nM, 100 μM), Figure S6: The resistance of the biosensor with the different Ag(I) concentrations (100 pM, 100 nM, 100 μM).
