# Supplementary material for: Label-Free Biosensor Using a Silver Specific RNA-Cleaving DNAzyme Functionalized Single-Walled Carbon Nanotube for Silver Ion Determination
Source: Nanomaterials (Basel). 2018 Apr 20;8(4):258. doi: 10.3390/nano8040258 (PMC5923588; doi:10.3390/nano8040258)
Supplement: Supplementary file 1 [file nanomaterials-08-00258-s001.pdf]

Supporting Information

Label-free biosensor using a silver specific RNA-cleaving DNAzyme functionalized single-walled carbon nanotube for silver ion determination

Hui Wang [1], Yang Liu[2] and Gang Liu [2, *]

1. Key Laboratory of Modern Precision Agriculture System Integration Research, Ministry of Education and Key Laboratory of Agricultural Information Acquisition Technology, Ministry of Agriculture China Agricultural University, Beijing 100083, P.R. China. wanghui_lunwen@163.com (H. W.);
2. Xi'an Jiaotong University, School of Electronic and Information Engineering, Xi'an 710049, P.R. China. yliu@sei.xjtu.edu.cn (Y. L.)

* Correspondence: pac@cau.edu.cn ; Tel.: +86-10-6273-6741

Keywords: Biosensor; Field effect transistor; Single carbon nanotube; Silver ion, Biosensor; DNAzyme

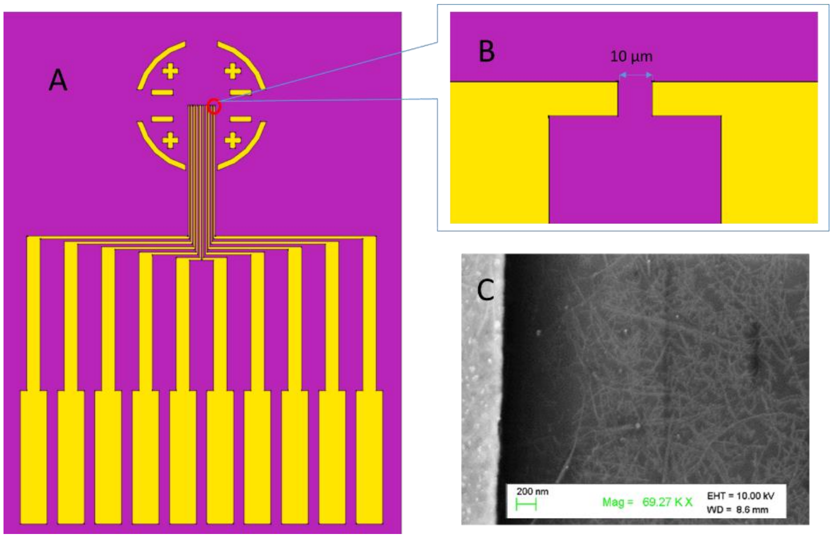

Figure S1. SEM image of SWNT networks produced by APTES-assisted assembly technique.

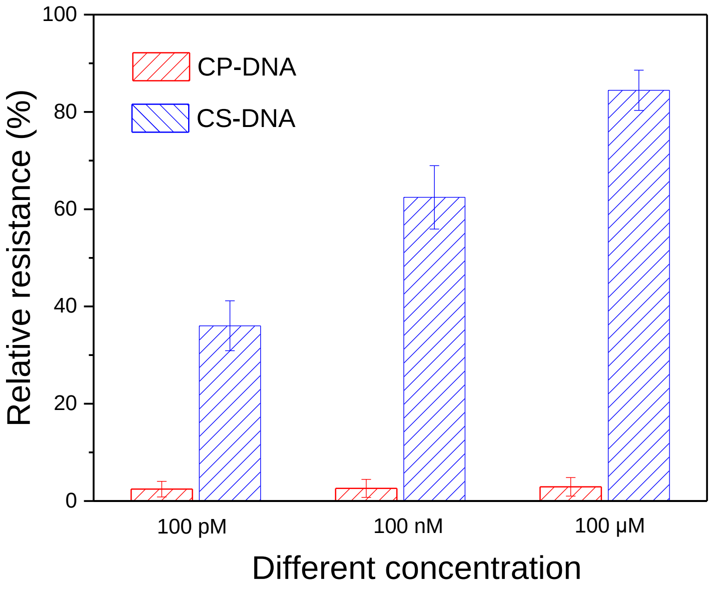

Figure S2. Effect of the 'A' base type of the Ag cleavage junction on the performance of the biosensor with the three Ag(I) concentrations (100 pM, 100 nM, 100 μM).

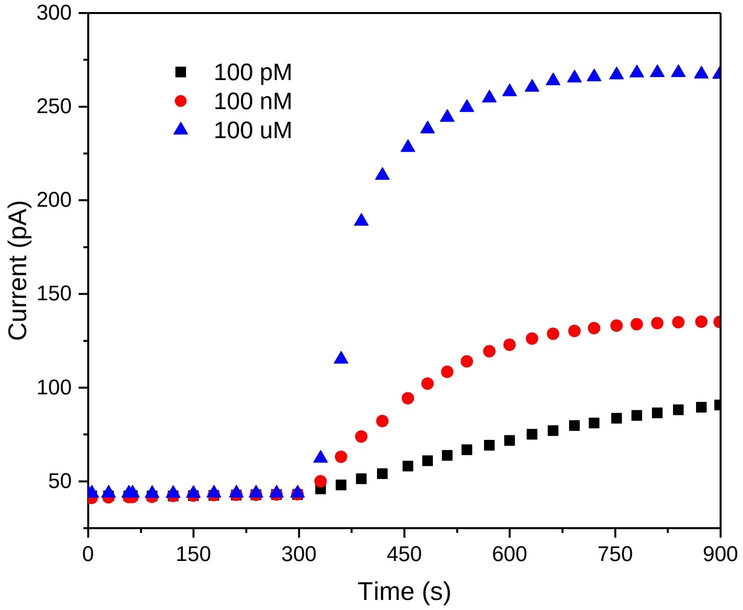

Figure S3. The relationship between current and incubation time on the performance of the biosensor with the three Ag(I) concentrations (100 pM, 100 nM, 100 μM) at V_G=0 V.

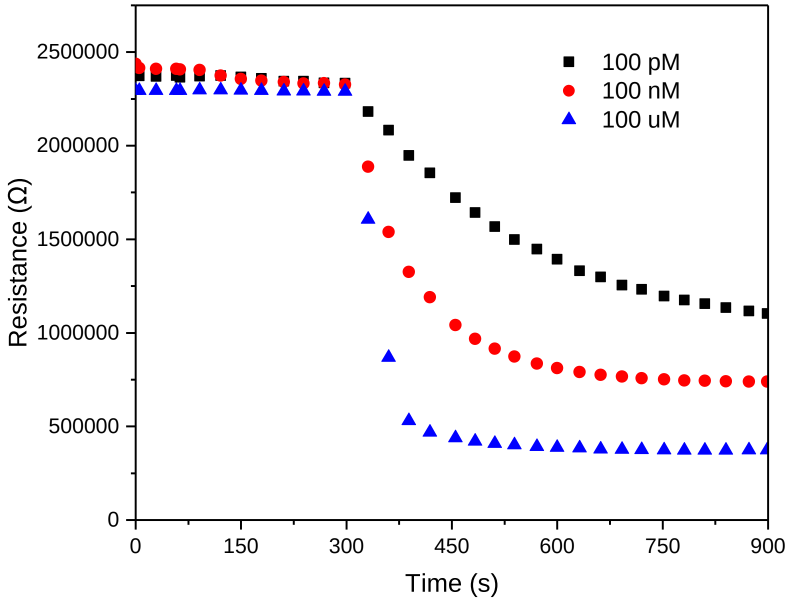

Figure S4. The relationship between resistance and incubation time on the performance of the biosensor with the three Ag(I) concentrations (100 pM, 100 nM, 100 μM) at V_G=0 V.

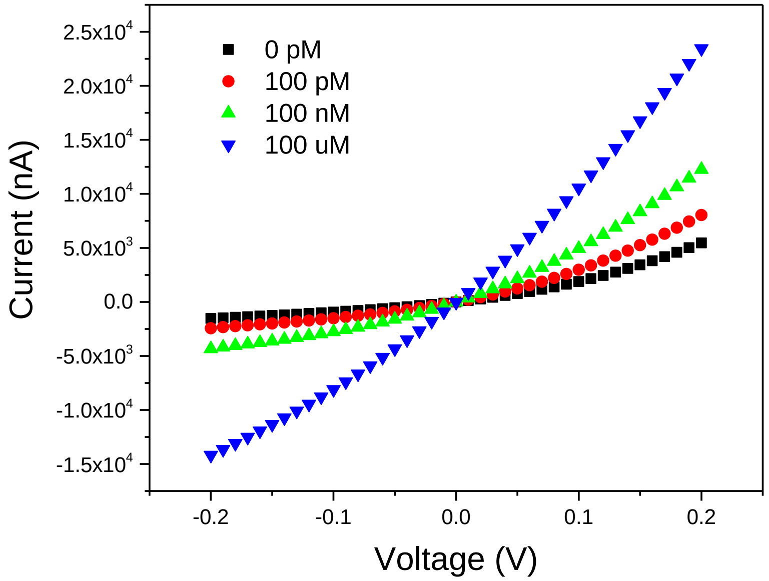

Figure S5. The effect of the voltage ranging from -0.2 V to 0.2 V on the performance of the biosensor with the three Ag(I) concentrations (0 pM, 100 pM, 100 nM, 100 μM).

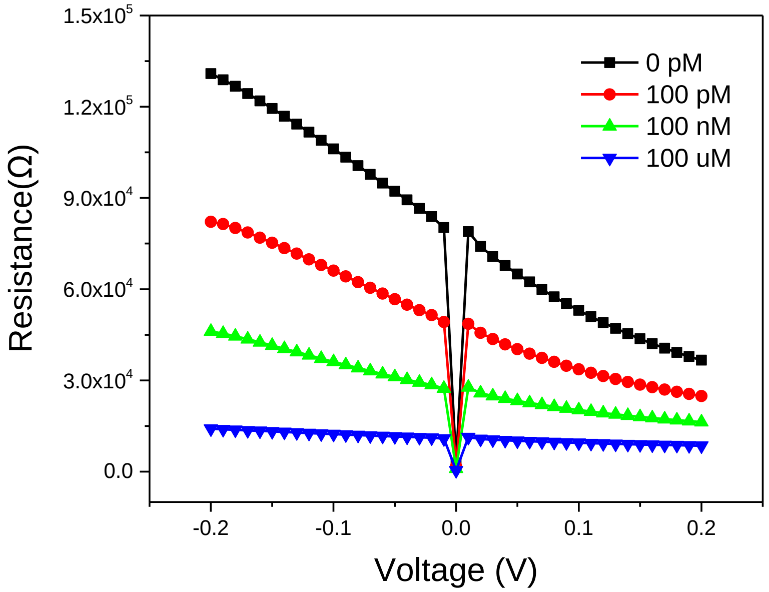

Figure S6. The resistance of the biosensor with the different Ag(I) concentrations (100 pM, 100 nM, 100 μM).
